# Relation of the Blood to Vaccinal Immunity

**Published:** 1877-11

**Authors:** 


The Relation of the Blood to Vaccinal Immunity.—The author has endeavored to prove experimentally what vehicle conveys to the vaccinated organism immunity from small-pox. Acting upon the probability that it was the blood, he introduced beneath the skin of children who had not been vaccinated a drop of blood from vaccinated children. As the length of time after vaccination might have an effect upon its power of inoculation, he took blood at various periods after vaccination from the first to the fortieth day. In spite of all, the result of the collective experiments, thirty-five in number, was absolutely negative. In two cases the blood was taken from the immediate vicinity of the pustule, but this blood was also not inoculable. In five cases trial proved that the vaccinated children were susceptible to the influence of the virus. As it was possible that the failure was due to the small amonnt of inoculated blood used, the author tried transfusion of blood in animals. Two hundred and fifty grammes of blood was withdrawn from the jugular vein of a heifer, upon which, at the sixth day after vaccination, the pustule was perfect, and transferred into another heifer three months old. During the next five days nothing abnormal was seen in the condition of the animal. Fourteen days later sixty inoculations were made upon the chest with fresh vaccine; nevertheless not a single pustule was developed, while another heifer, inoculated with the same virus, showed a splendid eruption. It follows, therefore, that under certain circumstances the blood is the vehicle of the material which gives immunity to the organism. The author has observed no indication upon the surface of deeper alterations produced by the transfusion, except the failure of vaccination. The objection which might be made to the experiment, that the animals experimented upon were protected by previous inoculation, is according to the experience of Chambon, exceedingly improbable, as in eight years' practice upon heifers, there has never occurred a single case in which the inoculation was without result.—*Raymaud Comptes Rend.*, 84.—*Allg. Med. Cent.-Zeit.*

